# Access to Meckel’s cave for biopsies of indeterminate lesions: a systematic review

**DOI:** 10.1007/s10143-020-01247-w

**Published:** 2020-02-10

**Authors:** E Suero Molina, JM Revuelta Barbero, C Ewelt, W Stummer, RL Carrau, DM Prevedello

**Affiliations:** 1 Department of Neurosurgery, University Hospital Münster, Albert-Schweitzer-Campus 1, A1, 48149 Munster, Germany; 2 Department of Otolaryngology-Head and Surgery, The Ohio State University, Wexner Medical Center, Columbus, OH USA; 3 Department of Neurological Surgery, The Ohio State University, Wexner Medical Center, Columbus, OH USA

**Keywords:** Meckel’s cave, Approaches, Indeterminate lesions, Endoscopic endonasal approach, Skull base surgery

## Abstract

Accessing Meckel’s cave (MC) is surgically challenging. Open approaches are complex and often correlated with high morbidity. Endoscopic approaches emerged in the last decade as feasible alternatives to open approaches, especially for sampling indeterminate lesions. This article first analyses available routes to approach Meckel’s cave and presents furthermore an illustrative case. We conducted a systematic review and reported according to the guidelines for preferred reporting items for systematic reviews and meta-analyses (PRISMA). Various surgical approaches identified through the search are evaluated and discussed in detail. Additionally, we report on a case of woman with a lesion in MC, which was accessed through an endoscopic transpterygoid approach subsequently diagnosed as a diffuse large B cell lymphoma. Our search delivered 75 articles that included case reports (*n* = 21), cadaveric studies (*n* = 32), clinical articles (*n* = 16), review of the literatures (*n* = 3), as well as technical notes (*n* = 2) and a radiological manuscript (*n* = 1). Open routes included lateral approaches with many variations, mainly intra- and extradural pterional approaches and anterior petrosal, as well as a retrosigmoid intradural suprameatal and a lateral transorbital approach. Endoscopically, MC was reached via approaches that included transpterygoid, transorbital or infraorbital fissure routes. Percutaneous approaches, e.g. through the foramen ovale, were also described. Multiple surgical approaches to MC are currently available. Their different characteristics as well as individual patient factors, such as clinical history and the localization of the disease, have to be considered when choosing a surgical corridor. Studies included in this review highlight the endonasal endoscopic transpterygoidal technique as an excellent corridor for biopsies in the ventral MC.

**Electronic supplementary material:**

The online version of this article (10.1007/s10143-020-01247-w) contains supplementary material, which is available to authorized users.

## Introduction

Named after Johann Friedrich Meckel, a German anatomist, the cavum meckeli, also known as Meckel’s cave, trigeminal cave or cisterna *trigeminalis*, is a region with a complex neurovascular array and, therefore, anatomically speaking, presenting a surgical challenge.

Meckel’s cave is located at the petrous apex between two dural layers originating from the floor of the middle fossa and dividing at the trigeminal notch, complemented by the dura propria of the posterior fossa [1–3]. Laterally, Meckel’s cave is limited by a meningeal layer covering the temporal lobe, whereas its medial wall separates the intercavernous carotid and sphenoid body from the trigeminal nerve [3]. Infero-medially, Meckel’s cave meets the bony part of the temporal fossa, as well as the petrous carotid canal [4, 5].

Due to Meckel’s cave location interfacing the posterior and middle fossae, lesions can spread between compartments, and thereby requiring access through a multi-corridor surgery.

External approaches have been historically applied to access this region [6–9]. The introduction of rod-lens-endoscopes allowed for minimal-access routes to the sellar and parasellar region affording exposure of the anterolateral and inferior portion of Meckel’s cave [5], thus improving cosmetic results and potentially decreasing surgical morbidity [10]. Since these approaches offer a wide visualization of relevant structures through a small surgical window, both biopsies and larger resections are feasible [5, 7, 11–14].

In lesions where the junction of clinical, radiological and laboratory data are not conclusive to suggest a therapy algorithm, tissue sampling might be imperative to establish a definitive diagnosis and treatment plan. This study aims to analyze different surgical approaches to reach Meckel’s cave for tissue sampling of such indeterminate lesions.

## Methods

### PRISMA literature search protocol

In this article, we searched and reported according to guidelines established by Preferred Reporting Items for Systematic Reviews and Meta-Analysis (PRISMA statement). The protocol included articles published until November 2018 without omission of earlier dates. Terms for searching title and abstract were “Meckel’s cave” and “biopsies”, “Meckel’s cave” and “biopsy”, “Meckel’s cave” and “endoscopic”, “Meckel’s cave” and “approach”, “Meckel’s cave” and “door”, “Meckel’s cave” and “access”, and “Meckel’s” and “resection”. Articles delivered by the initial search were screened for duplicates and non-English abstracts. After eliminating these studies, abstracts were screened and relevant full-texts were evaluated. The search was conducted according to the outlined protocol using commercially available software (Endnote X7, Thompson Reuters, Carlsbad, California, USA).

## Results

The initial search yielded 271 articles. First, duplicates (*n* = 89) and non-English abstracts (*n* = 25) were removed; thereafter, 157 abstracts were screened for relevance resulting in the full-text evaluation of 112 articles. Subsequently, 75 articles were identified for our qualitative synthesis and included case reports (*n* = 21) [15–35], cadaveric studies (*n* = 32) [3, 6, 7, 10, 13, 36–60], clinical articles (*n* = 16) [2, 5, 8, 9, 16, 61–71], review of the literatures (*n* = 3) [72–74], as well as technical notes (*n* = 2) [75, 76] and a radiological manuscript (*n* = 1) [77]. These outlined articles were published between February 1978 and November 2018. Additional citations were included when relevant. Rather than describing each approach in detail, the study aimed to outline essential information. The authors refer to the respective publications for further details.

### Approaches to Meckel’s cave in the literature (Fig. 1)

#### Antero-medial

##### Extended endonasal endoscopic-assisted approach (with illustrative case)

Endoscopic approaches to the skull base are promising due to improved visualization and reduced morbidity in comparison to external approaches. Furthermore, they lack the need for crossing cranial nerves and vessels [7]. In a sufficiently pneumatized sinus, a wide sphenotomy will already provide access to the anterior portion of Meckel’s cave [12, 58, 74]. However, this might not be sufficient if targeting the lower lateral skull base [53]. For more extended visualization of Meckel’s cave, several approaches have been reported in the literature:

**Fig. 1:**
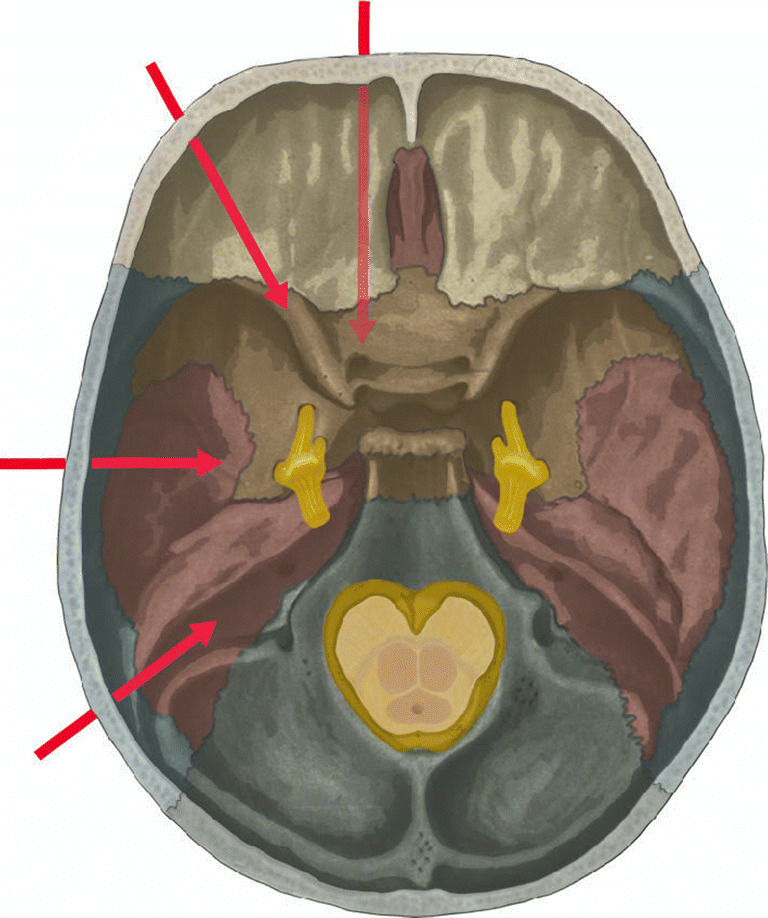
Skull base illustration highlighting available routes to Meckel’s cave. These can be divided in antero-medial, antero-lateral, lateral and posterior (red arrows from upper right to lower left, respectively)

#### Transpterygoid approach

The transpterygoid approach extends the endonasal corridor to address tumors in the middle and posterior fossa [77]. Removing the medial aspect of the pterygoid process base can already provide sufficient access to Meckel’s cave [17]. An ipsilateral middle turbinectomy and uncinectomy, followed by a posterior ethmoidectomy and a wide sphenotomy are normally performed [5]. Lateralization of the inferior turbinate and the contralateral middle turbinate by out-fracturing their bony attachment can increase working space [78]. Additionally, a wide maxillary antrostomy with lateral exposure of the infraorbital fissure with its neurovascular structures can increase the panoramic view of the skull base [5, 70]. The infraorbital nerve delineates the pterygopalatine fossa (PPF) laterally increasing orientation during the procedure.

The Vidian nerve and artery have become an important landmark to identify the inner anterior genu of the petrous segment of the ICA, for improving depth perception during the surgical procedure and avoid injury of the ICA [7, 11, 38, 77–79]. The Vidian neurovascular bundle is identified where the medial pterygoid plate meets the floor of the sphenoid sinus, in average 12.78 mm (range 9.4–15.8 mm) from the midline [77]. This bundle can be either coagulated and divided [5, 78], facilitating lateralization of the PPF content, i.e. int. max. art, V2 and pterygopalatine ganglion [7], or preserved if approaching strictly superior to the Vidian nerve [14, 70]. The Vidian nerve, however, transports sympathetic and parasympathetic fibers important for lacrimation [7]. Hence, Vidian nerve injury can impair lacrimation in the ipsilateral eye. The complex relationship of the ICA at the posterior limit of this route with the Vidian nerve and the quadrangular space is essential for the safety of these surgeries [44, 52, 80]. Skeletonizing the ICA is only required if mobilization is needed for posterior access [7].

A quadrangular space delineated by the ICA medially and inferiorly, the V2 nerve laterally, which is superolateral to the ICA [7], and the abducens nerve with the CS superiorly, provides access to Meckel’s cave [79]. The ICA course should be carefully studied to define the feasibility of this approach [13] and avoid injury while drilling.

To prevent injury of the abducens nerve, V2 should not be crossed superiorly and intraoperative electrophysiological monitoring should be applied [5, 39]. Furthermore, drilling near the petrous apex can induce thermal injury of the VI^th^ nerve [39]. As an orientation, the superior part of the lacerum segment of the ICA correlates with the dural entry point of the VI^th^ nerve posteriorly [39]. Access to the petrous apex requires bypassing the ICA [50]. This route carries limitations for lesions extending into the posterior fossa. However, lesions in the anterior-medial and inferior portion of Meckel’s cave can be easily accessed.

#### Illustrative case

**Fig. 2:**
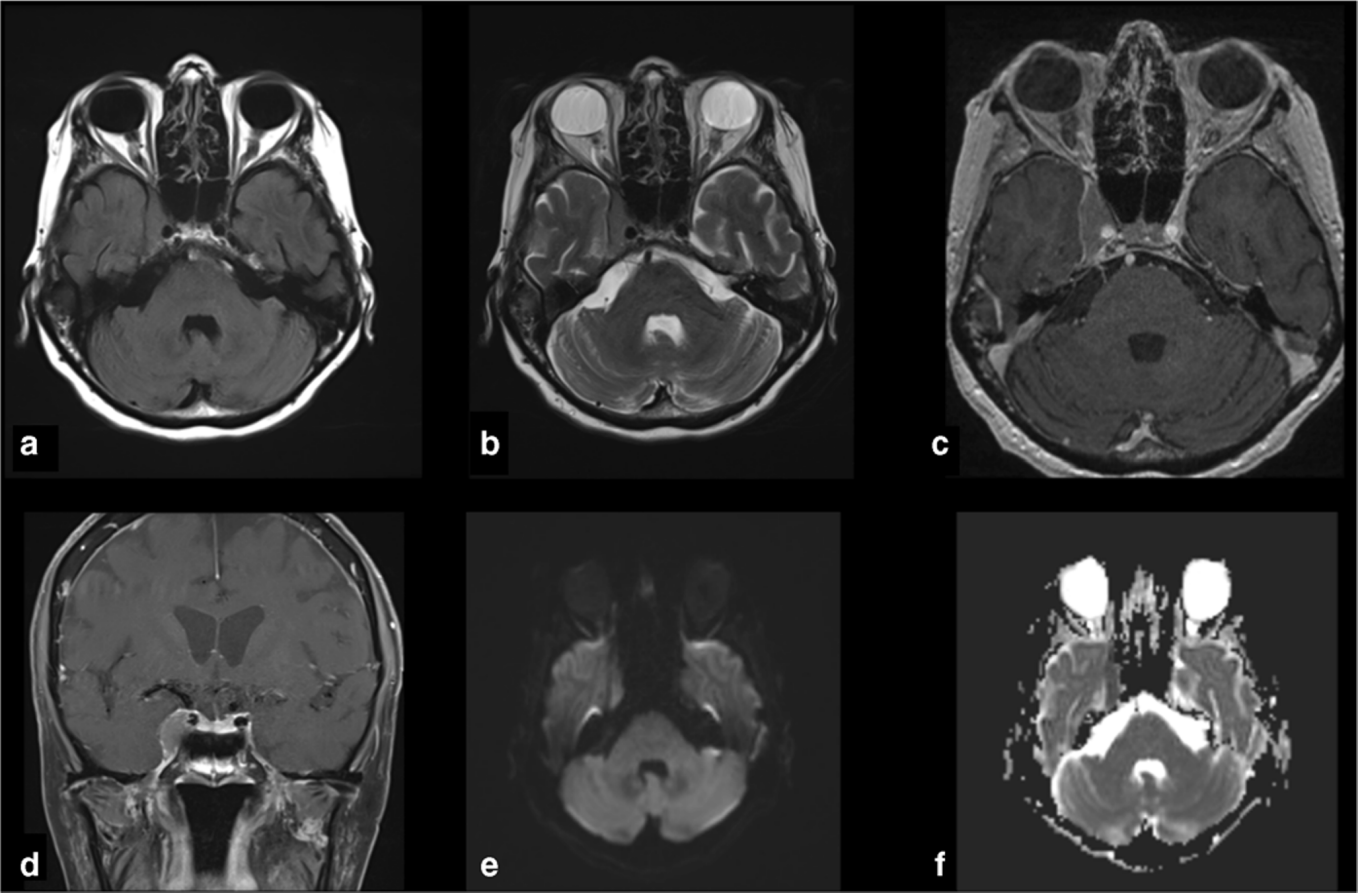
Preoperative imaging of a 78-year-old female patient harboring a lesion in Meckel’s cave. No hyperintensity was observed in the **a** FLAIR- or **b** T2-weighted scan. There was however contrast-enhancement demonstrated in the **c** axial and **d** coronal T1-weighted imaging. No diffusion restriction could be seen in the **e** DWI, but hypercellularity was demonstrated in the **f** ADC-sequence

A 78-year-old female patient presented to the James Comprehensive Skull Base and Pituitary Center, Columbus, Ohio, USA due to a progressive cranial nerve VI palsy, retro-orbital pain and proptosis on the right side. The patient had a pre-medical history of non-Hodgkin lymphoma with a newly identified Meckel’s cave mass (Fig. 2).

**Fig. 3:**
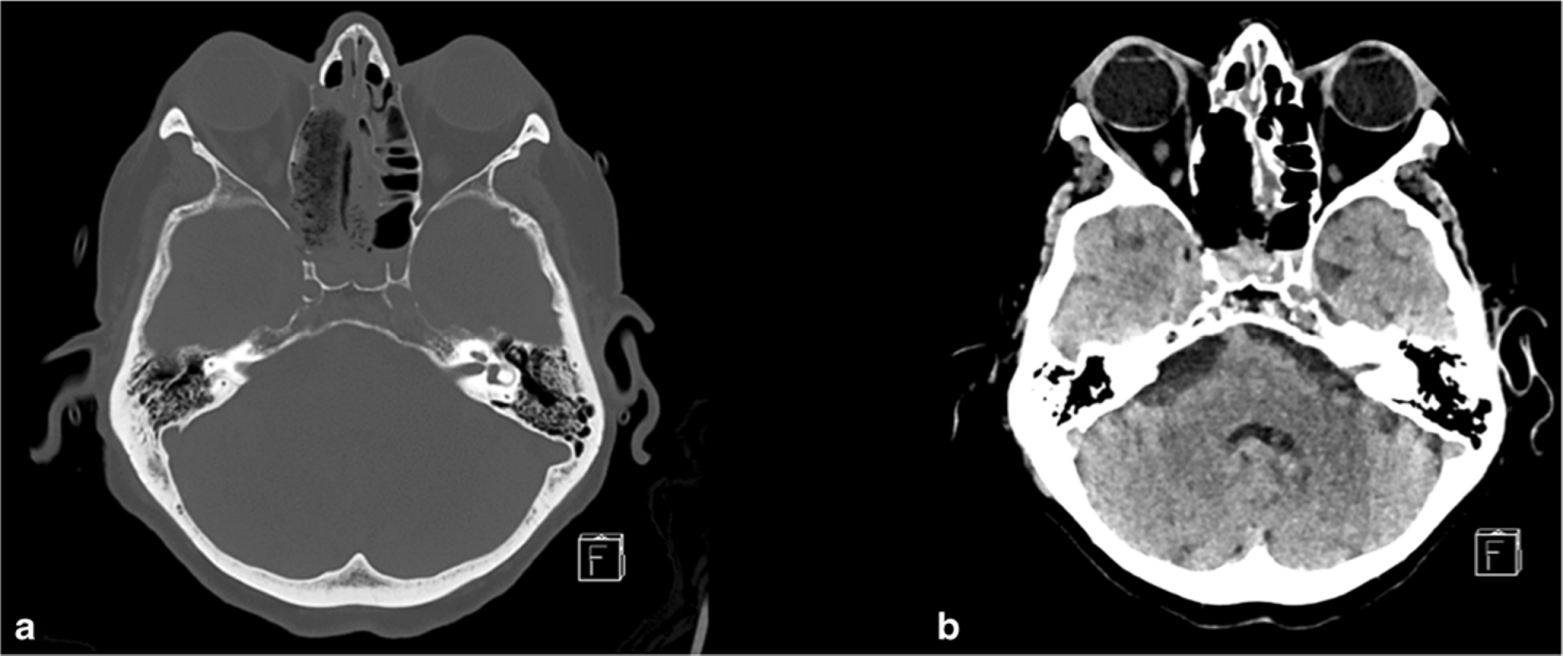
Postoperative CT-imaging after endonasal endoscopic transpterygoidal approach to Meckel’s cave with **a** bony and **b** tissue windowing demonstrating the bone access to Meckel’s cave, as well as the defect after extended endonasal transpterygoidal approach with right side turbinectomy, uncinectomy, posterior ethmoidectomy and wide sphenotomy

Intending biopsy, the lesion in the right Meckel’s cave was accessed through an extended endonasal endoscopic-assisted transpterygoidal approach (Video 1; Fig. 3). A free mucosal graft was employed for reconstruction of the skull base defect (from the right middle turbinate). The mass was later diagnosed as a diffuse large B cell lymphoma with double expressor (C-MYC+ and Bcl-2+). More in detail, the tumor was classified as germinal center type given CD10 positivity, in the presence of strong MUM-1 staining; these cases have been reported to follow a more aggressive clinical course. The patient postoperative recovery was uneventful. The right retro-orbital pain diminished and double vision improved. There was no sign of new facial numbness or paresthesia.

#### Transmaxillary

As an alternative to the transpterygoid approach, removing the posterior maxillary sinus will also lead to Meckel’s cave [57, 60, 71]. Zhang et al. [60] described an endoscopic access through the inferior orbital fissure. They describe an approach lateral to the middle turbinate, where—after performing an uncinectomy and medial maxillectomy—the infraorbital neurovascular bundle could be transposed inferiorly, providing space for drilling the anterior portion of the sphenoid wing lateral to the foramen rotundum and gain anterolateral access to Meckel’s cave. Previously, Bai et al. [38] described a similar approach reaching the anteromedial portion of Meckel’s cave. As a variance, a sublabial transantral route might lessen the need of removing the anterior sphenoid wall [10, 57]. An anterior antrostomy through a sublabial incision provides access superior to the alveolar ridge. Through the canine fossa, an anterior antrostomy is performed, and dorsal to the maxillary sinus, the infraorbital nerve and subsequently V_2_ are dissected to reach Meckel’s cave. Access to Meckel’s cave will, however, be limited [57]. These articles discuss the advantage of less manipulation to the structures within the pterygopalatine and infratemporal fossa, as well as within the cavernous sinus and the petroclival carotid, arguing to cause less trauma and providing a safer approach. However, the nasolacrimal duct needs to be transposed and the infraorbital neurovascular array with emerging nerves, i.e. the zygomatic or lacrimal nerve, manipulated; clinical sequela can therefore be of relevance.

#### Transorbital

Two cadaveric studies outlined the anatomical and technical nuances of the lateral endoscopic orbital route to access Meckel’s cave [47, 54]. This corridor was used to reach the middle fossa and the lateral ventral skull base and Meckel’s cave. The skin incision was made either in the superior eyelid [54], or at the inferior orbital rim [47]. Accessing through the lateral orbit and between the superior and inferior orbital fissure, a triangular [47] or trapezoid [54] shape craniotomy was described. Finding an extradural space superior to V_2_ and by further drilling posterior to V_3_, Meckel’s cave could be accessed in an oblique anterosuperior way. The trigeminal nerve could be visualized from the cisternal portion in the posterior fossa until its division in V_1_-V_3_ [54]. Additionally, the superior and posterior CS, as well as the superior petrosal sinus, were identified [47]. The region medial to V1 and posterior to the ICA appears an anatomical limitation of this approach. This approach targets the anterolateral and superior region of Meckel’s cave. Jeon et al. demonstrated in a recent series of nine patients, seven of them with diseases involving Meckel’s cave, the feasibility of this novel technique [65]. Even though the sample size is small and the follow-up time was short, the authors reported low morbidity with a single patient suffering of ptosis, which eventually recovered after 6 months. Gross total resection was achieved in seven of nine patients.

#### Percutaneous

Transforamen ovale image-guided approaches are regularly used for rhizotomy in the context of atypical trigeminus neuralgia [81]. In 1997, Sindou et al. described the percutaneous approach to Meckel’s cave for biopsies of indeterminate lesions, based on their trigeminal thermocoagulation experience [82]. CT-guidance might increase safety [35]. Even though this approach appears feasible, highly vascularized tumors can be of high risk for hemorrhagic complications. Tumor consistency is further important, since hard tumor tissue is difficult to aspirate through a needle [18]. Messerer et al. reported contamination of sample tissue with fat, CSF or blood, leading to diagnostic difficulties [83]. Drainage of arachnoid cysts, however, has been reported as feasible [21]. In the further course, an endoscopic transforamen ovale approach and observation of Meckel’s cave were reported [48]. However, vision was restricted by the lack of space and mobility of the endoscope and by oozing from small veins. Variations of the internal maxillary artery can be of relevance and should be carefully evaluated preoperatively [37]. Nevertheless, this approach should be kept in mind as an option to manage indeterminate lesions, as it is associated with low morbidity and not all pathologies require a surgical therapy [18]. Hence, unnecessary open surgery could be avoided.

#### Anterolateral and lateral approaches

Anterolateral approaches consist of frontotemporal or orbitozygomatic approaches with intra- or extradural corridors. Major drawback from this corridor is the need of temporal lobe retraction, especially when targeting the inferior part of Meckel’s cave. Augmenting this approach with an orbitozygomatic removal, as well as the dissection of the temporal muscle [31], can decrease retraction of the temporal lobe [62, 84]. By opening the sylvian fissure, the view into Meckel’s cave and cavernous sinus (CS) can be extended [4, 22, 85].

When approaching from the extradural space, elevation of the middle fossa dura is limited medially due to attachment to the V_3_-Branch and the CS [6]. Furthermore, the meningeal medial artery at the foramen spinosum and V_3_ at the foramen ovale can be identified before exiting the skull base [6]. Skeletonizing the superior orbital fissure and foramen rotundum and ovale will help delineate the plane of dura elevation [56].

A frontotemporal extra-/interdural approach (Dolenc’s approach [86]) can avoid exposition of the temporal lobe and enable exposure of the trigeminal ganglion [59, 62]. From lateral, Meckel’s cave is best identified around 7.5 mm medial of the foramen spinosum and just posterior to the foramen ovale [6]. The meningeal dura is kept covering the temporal lobe and the entire lateral surface of Meckel’s cave is exposed.

Most of these approaches were created to excise schwannomas or meningiomas in the petroclival or parasellar region [67, 68]; they provide wide access to the superior lateral Meckel’s cave in exchange of higher risk of morbidity.

#### Subtemporal transpetrosal-transtentorial approach with anterior petrosectomy (Kawase-Shiobara approach)

Kawase et al. described an anterior petrosectomy by removing the bone ventral to the IAM. This route can target pathologies in the upper petroclival region, Meckel’s cave and brainstem [1, 55]. There is a risk of IV^th^ nerve injury if the tentorium is incised with the aim to improve access to the infra- and supratentorial petroclival region [50]. However, this incision is not necessary in most cases.

Great superficial petrosal nerve (GSPN) is identified as an important landmark during anterior petrosectomy [66, 87]. Drilling anterior to the bone of the internal acoustic meatus can cause damage to the cochlea [6]. The approach will be limited by the lower edge of the porus trigeminus, inferior petrosal vein and the petrous ICA [6, 87]. Removal of the Kawase triangle is only essential when exposure of the ventral brainstem and clivus is needed [1, 88]. Lesions from Meckel’s cave with lateral or posterior fossa extension can be reached through this approach.

Excessive retraction of the temporal lobe should be avoided, since this could damage the V. Labbe [89], or induce seizures [90]. Further risks are cranial nerve or vascular injury, CSF leak and damage to the intrapetrous otologic structures, i.e. geniculate ganglion (facial palsy) or cochlea (hearing loss) [91]. To avoid brain stem and cerebellum edema, the superior petrosal vein should be sheltered [92].

### Postero-lateral

#### Retrosigmoid intradural suprameatal approach

The retrosigmoid approach has been discussed as a route to the petroclival region since the beginning of the neurosurgical era [93]. As a modification of the retrosigmoidal approach for lesions extending to the middle fossa, postero-lateral approaches interconnect both the middle and the posterior cranial fossa [8, 72]. Even though a semi-sitting position is commonly used, park-bench positioning might reduce the risk of venous air embolism. The surgical corridor consists of a retrosigmoid approach with additional drilling of the suprameatal bone prominence and the posterior portion of the petrous apex [51]. The latter step can further expose the lateral trigeminal nerve by an average of 10 mm (range 6–13 mm) [3, 36, 41]. The trigeminal impression represents the anterior limit of the bony resection [3, 8].

Optionally, the tentorium can be divided above the V^th^ nerve for further access to the middle fossa. Drilling the suprameatal tubercle in a pyramidal shape, with the base towards the trigeminus nerve, can avoid injuries to both superior and posterior semicircular canal, as well as to the common crus of both canals [36]. Additionally, endoscopic-assistance with 0- and 30-degree endoscopes has been reported to be feasible for identifying deep-seated neurovascular structures [36, 45, 94]. The trochlear nerve can be identified in the cisterna ambiens medially under the tentorium before trespassing dorsal to the posterior clinoid process [36]. The abducens nerve regularly lies underneath and medial to the IV^th^ cranial nerve traveling to the clivus before it enters into the Dorello’s canal and towards the CS [36].

An advantage of this approach is that no blind tentorial splitting or petrosectomy is needed. However, risks that merit mention are cranial nerves and vessels injury, e.g. superior petrosal vein, sigmoid and transverse sinus, anterior and posterior inferior cerebellar artery, and potential injury through a cerebellum and brainstem retraction.

Other postero-lateral approaches with posterior transpetrosal modifications and presigmoid access can be retrolabyrinthine, translabyrinthine or transcochlear maximizing the petroclival window. Those bear, however, a greater risk of hearing impairment or facial palsy and normally do not provide enough exposure of Meckel’s cave [95–97].

A partial labyrinthectomy petrous apicectomy combining potential advantages of retro- and translabyrinthine corridors has also been described as a modification of these approaches [40], but is likely to be too invasive if solely used for biopsies.

#### Other approaches

A midface degloving approach has been also described to access the anterior skull base [16]. However, approaching Meckel’s cave required sacrifice of the maxillary nerve and the approach itself appears to be invasive in terms of scaring.

## Discussion

Historically, approaches to Meckel’s cave have been divided in anterolateral, lateral and posterolateral, comprising frontotemporal extra- or intradural, orbitozygomatic, subtemporal anterior petrosal, presigmoid posterior petrosal and suboccipital approaches [5, 43]. More recently, the anterior-medial route with the help of rod-lens endoscopy is providing minimal-invasive access to this region [70].

Expanse of Meckel’s cave is predominantly determined by the size of the trigeminal ganglion. Its width correlates with the medial to lateral dimension, and its length with the distance from the trigeminal porus to the anterior edge of the trigeminal ganglion [6]. The dura carpeting the floor of the middle fossa, splits at the trigeminal notch in two-sheets that build the layers covering Meckel’s cave and exit through the porus trigeminus posteriorly towards the posterior fossa [3]. The lateral dural wall is formed by the tentorium, whereas the cavernous sinus and the petrolingual ligament, as a continuity of the carotid canal periosteum, constitute the medial wall [2]. Meckel’s cave interconnects the middle and posterior fossa and tumors can spread through these regions [98], creating unphysiological spaces, that should be considered for planning approaches. Content of Meckel’s cave includes the Gasserian ganglion and postganglionic trigeminal rootlets lying in the trigeminal cistern [6]. In most cases, a thin bony lamina is found between trigeminal ganglion and internal carotid artery (ICA), but dehiscence occurred in up to 30% of analyzed specimens in a recent study [6]. The abducens nerve travels in the posterior inferior cavernous sinus, in close vicinity inferior and medial to the TG and Meckel’s cave [43]. Oculomotor and trochlear nerve run superior to the trigeminal ganglion (~ 5–6 mm) [6].

### Differential diagnosis of lesions in Meckel’s cave

#### Decision-making

We have highlighted the anatomic limitations, technical nuances and potential advantages of each route. Especially for biopsies, a small window towards the lesion might be sufficient. A large portion of tumors in Meckel’s cave will have an extra-/intradural location [67], intradural approaches are often not needed. However, case selection has been to date discussed according to the radiological appearance of lesions and their relationship to the dural sheets [2], which is not always feasible when dealing with the indeterminate lesions discussed in this article (Table 1). If a further resection is required, the feasibility of each approach need to be evaluated according to the specific anatomical situation.

**Table 1:** Differential diagnosis of lesions within Meckel’s cave

Benign	Meningioma [61]
	Benign Schwannoma [61]
	Benign melanotic schwannoma [99]
	Xanthoma [100]
	Lipoma [61]
	Neuromuscular hamartoma [33]
	Hemangioblastoma [27]
	Cavernous hemangioma [63]
	Pituitary adenoma [63]
Malignant	Malignant peripheral nerve sheet tumor [63]
*Primary*	Nasal glioma [25]
	Atypical teratoid-rhabdoid [20]
	Intradural chordoma [15]
	Chondrosarcoma [63]
	Paraganglioma [30]
	Rhabdomyosarcoma [63]
*Metastatic*	
	Neuroendocrine carcinoma [63]
	Adenoid cystic carcinoma [5]
	Malignant melanotic schwannoma [23, 61, 101]
	Squamous cell carcinoma [63]
	Adenocarcinoma [63]
Inflammatory	Sarcoidosis [19, 69]
	Amyloidoma [22, 24, 28]
	IgG4 disease [63]
	Necrotizing granulomatous inflammation [63]
	Inflammatory pseudotumor [63]
Hematologic malignancies	Primary malignant lymphoma [26]
	Multiple myeloma [32]
	NK/T lymphoma [63]
	Diffuse B-cell lymphoma*
	Plasmacytoma [63]
	Marginal zone lymphoma [63]
	Chronic eosinophilic leukemia [63]
	Lymphoplasmocytic lymphoma [63]
	Non-Hodgkin lymphoma [63]
Cystic	Arachnoid cyst [5, 21, 34, 61, 64]
	Epidermoid cyst [17, 29]
	Meningoceles [102]

*Illustrative case report presented in this article

Tumor consistency is important, as hard, fibrous tumors might require a wider exposure, than cystic or soft tumors, where a narrow approach could be sufficient [49]. Thorough imaging with MRI and CT is therefore essential. Cavernous sinus invasion is suspicious of meningioma or hemangiopericytoma [2]. Hence, an approach where further resection is possible should be chosen. Tumor extension might be the most important factor towards surgery planning of these lesions. Patient’s morbidity and age might steer surgeons towards only biopsing lesions, or simply decompressing important neurovascular structures. Finally, the surgeon’s experience will always lead the discussion. The available equipment and capacities of each institution will also play a role in decision-making. However, whatever skull base approach is applied, it has to be studied in detail and performed with experience to achieve excellent results.

The endoscopic endonasal approach provides safe access to Meckel’s cave [7], with the transpterygoidal route supported by the most clinical reporting. If needed, the possibility for further tumor removal is given. The discussed approaches should, however, be seen as complementary and not competitive [73], since each one of them carries its own risks and advantages. Even though newly described endoscopic approaches appear promising, clinical experiences remain to be reported and an advanced anatomical knowledge of the neurovascular array within and surrounding the skull base is of utmost priority to assure safety during and after procedures. Hence, outcome reports from clinical series are further needed and case selection should be thoroughly discussed (Table 2).

**Table 2:** Outline of available approaches highlighting advantages and risks as well as potential complications

		Portion MC access	Advantages	Structures at risk/complications	Limits
Anterio-medial	Transpterygoid	Antero-medial inferior	No brain retraction	CSF leak, vidian nerve/artery, corneal keratopathy, internal carotid artery	Content infratemporal fossa, region lateral and posterior to the GG
	Transantral/-maxillary	Antero-lateral	Less ICA/PPF manipulation	Oro-antral fistula, nasolacrimal duct	Posterior fossa
	Transorbital	Superior and anterolateral	Limited temporal retraction, no manipulation of PPF content	Orbital content, cranial nerves, M. levator palpebrae, CSF leak and pulsatile exopthalmus	Region medial to V_1_, posterior to ICA
	Percutaneous	Foramen rotundum	Minimal invasive	Internal maxillary artery, cranial nerves, e.g. trigeminal and oculomotor nerves	Lack of surgical field visualization
Antero-lateral	Pterional and orbitozygomatic extension	Antero-lateral-superior	Standard skull base surgery approach	Cranial nerve injury (III, IV, VI), temporal muscle disruption/translocation brain retraction	Inferior portion of MC
Lateral	Anterior petrosectomy	Lateral, dorsal	Posterior fossa extension, if required	Brain retraction, wide craniotomy, tentorial division, otologic structures, superior petrosal vein	Lower edge porus trigeminus, petrous ICA, inferior petrosal vein
Posterior	Retrosigmoid-suprameatal	Dorso-medial	Small craniotomy	Cranial nerve injury (VII-VIII), sinus sigmoideus, cerebellum retraction	Middle fossa

*CSF* cerebral spinal fluid, *ICA* internal carotid artery, *GG* Gasserian ganglion, *MC* Meckel’s cave, *PPF* pterygopalatine fossa

#### Limitations

The amount of data available in the literature is by now immense. Our description is limited by the amount of data that is possible to include in a review article. We hope, however, to have provided a practical review of 360-degree approaches to Meckel’s cave, encouraging critical thinking and evaluation of lesions.

## Conclusions

This work, in an effort to shed light on the various routes to this region, provides an overview of the variance of approaches for reaching Meckel’s cave. Anatomical landmarks and their variations, as well as the disease extension, are essential when planning a surgical approach to Meckel’s cave. For lesions especially in the anterior, inferior and medial compartment of Meckel’s cave, the extended endoscopic endonasal transpterygoidal approach is an excellent approach for targeting these lesions [69]. Numerous of these approaches are complementary to each other. Hence, open approaches are to be selected when necessary.

It is clear that skull base surgeons should learn and study the different approaches and include them in their surgical armamentarium, to provide the safest route according to the underlying pathology.

## Electronic supplementary material

ESM 1 (MP4 96372 kb)
